# Optical biosensors using plasmonic and photonic crystal band-gap structures for the detection of basal cell cancer

**DOI:** 10.1038/s41598-022-09213-w

**Published:** 2022-03-28

**Authors:** Shiva Khani, Mohsen Hayati

**Affiliations:** Electrical Engineering Department, Faculty of Engineering, Razi University, Kermanshah, 67149-67346 Iran

**Keywords:** Optics and photonics, Physics

## Abstract

One of the most interesting topics in bio-optics is measuring the refractive index of tissues. Accordingly, two novel optical biosensor configurations for cancer cell detections have been proposed in this paper. These structures are composed of one-dimensional photonic crystal (PC) lattices coupled to two metal–insulator–metal (MIM) plasmonic waveguides. Also, the tapering method is used to improve the matching between the MIM plasmonic waveguides and PC structure in the second proposed topology. The PC lattices at the central part of the structures generate photonic bandgaps (PBGs) with sharp edges in the transmission spectra of the biosensors. These sharp edges are suitable candidates for sensing applications. On the other hand, the long distance between two PBG edges causes that when the low PBG edge is used for sensing mechanism, it does not have an overlapping with the high PBG edge by changing the refractive index of the analyte. Therefore, the proposed biosensors can be used for a wide wavelength range. The maximum obtained sensitivities and FOM values of the designed biosensors are equal to 718.6, 714.3 nm/RIU, and 156.217, 60.1 RIU^−1^, respectively. The metal and insulator materials which are used in the designed structures are silver, air, and GaAs, respectively. The finite-difference time-domain (FDTD) method is used for the numerical investigation of the proposed structures. Furthermore, the initial structure of the proposed biosensors is analyzed using the transmission line method to verify the FDTD simulations. The attractive and simple topologies of the proposed biosensors and their high sensitivities make them suitable candidates for biosensing applications.

## Introduction

Electronic devices^1,2^ are unable to fulfill all of the future’s expanding needs such as increasing the speed and reducing power dissipation. As a result, photonic integrated circuits such as photonic crystals (PCs)^3,4^ can be suitable candidates to replace electronic integrated circuits due to their higher computational speeds, higher information densities, and less noise. One-dimensional PC structures consist of a multilayer stack of insulator materials^5^. Appearance of photonic band-gap (PBG) is one of the most significant properties of these structures^6^. The dynamic shift of the PBG edge in PCs can be used to design various structures such as optical sensors^7^, switches^5^, and modulators^8^. Unfortunately, due to the diffraction limit of light, PC devices are not suitable structures for the realization of highly integrated optical circuits^5^.

Surface plasmon polaritons (SPPs) can be used to overcome the footprint problem^9^, by overcoming the diffraction limit^10^ and manipulating light at a sub-wavelength scale^11^. SPPs are the electromagnetic surface waves that propagate at the surface of the metal and insulator materials. In the low-frequency range, metal is treated as a perfect conductor. In the UV and visible region, metal can no longer be treated as a perfect conductor. It is because of its collective electrons excitation, which is called the plasmon. In this frequency range, metal can still be used to build a low-loss metal waveguide or metal–insulator waveguide. In these cases, the electromagnetic field takes the form of an evanescent field. Furthermore, the complex permittivity of noble metals such as gold and silver has a relatively larger real part than its imaginary part. Also, its real part is usually a large negative number in the near infrared region (NIR) and visible region. This optical property of metal causes the surface plasmon wave can propagate at the surface of the metal and insulator.

In addition, plasmonic structures have the integration capability with other electrical and microwave components^12–15^. Therefore, different metal–insulator–metal (MIM) plasmonic devices have been designed so far. Such devices include plasmonic filters^16–18^, splitters^19,20^, sensors^21–23^, demultiplexers^24,25^, slow light waveguides^26,27^, switches^28–31^, logic gates^32,33^, converters^34^, modulators^35,36^ and so on. It is worth mentioning that the main disadvantage of plasmonic structures^37,38^ compared to PCs^39,40^ is their higher absorption value which results in lower Q-factor. As a result, the combination of plasmonic and PC structures has been used to obtain a trade-off between various designing parameters in this paper.

Optical sensors^41–43^ are attracted important interest as their wide range of applications. One of the most significant applications of optical sensors is in the biomedical field. For example, such sensors can be used for cancer cell detections^42,44^, health care applications^45^, and blood component measurements^46^. Up to now, different approaches based on various configurations such as plasmonic^47^, PC^48^, graphene^49^, optical fiber topologies^50^, etc. have been adopted to design optical sensors. Since surface plasmons are sensitive to changes in the refractive index of the metal surface, this phenomenon can be used as a tool for optical sensing. Sensors designed using plasmonic structures are sensitive to refractive index changes so that by connecting the particle to the surface, refractive index changes will be detectable. In this method, the particle-to-surface connection is converted directly into a signal and does not require labeling, while in conventional optical sensors, chromophore colors are required. Recent progress in plasmon-based sensors has overcome the limitations of conventional optical sensors so that using such structures will enhance the sensitivity, optical stability, tunability, and usability of these sensors in the living environment.

The conventional configurations to design optical sensors are based on plasmonic Mach–Zehnder interferometer^51^, plasmonic square ring resonator^52^, plasmonic cross resonator^53^, rectangular plasmonic interferometer^54^, plasmonic triangular resonator^55^, one dimensional porous silicon PC sensor^56^, armchair graphene nano-ribbon^57^, and so on. All aforementioned sensor structures in the literature create conventional spectra like Lorentzian, Fano resonance, and electromagnetically induced transparency (EIT) spectra for sensing applications.

In this paper, two novel topologies have been proposed based on the combination of one-dimensional (1D) PC and MIM plasmonic configurations. In these structures, the PC topologies have been used at the central part of the sensor structures (between two MIM plasmonic waveguides) to create PBGs in the transmission spectra with sharp transient edges. These sharp edges increase the sensitivity of the proposed sensors. Accordingly, this spectrum type is a suitable choice for sensing mechanisms. Also, the tapering technique has been used in the second proposed topology to improve the matching between the plasmonic and PC sections. It is worth mentioning that the designed sensors can be used for the detection of the basal cell cancer. Today, cancer has spread worldwide in a way that has attracted the serious attention of researchers. Prompt and timely diagnosis of cancer is one way to determine the best treatment option. Cancer seems to be on the rise today due to environmental pollution, lifestyle, and nutrition. On the other hand, due to the high cost, lengthy and difficult treatment of cancer, early detection of cancer is very important for treatment.

The metal material of the substrate area in these structures is assumed to be silver, which is characterized by a well-known Drude model^58^. Meanwhile, the used insulator materials are air ($\varepsilon =1$) and GaAs (Palik model). The finite-difference time-domain (FDTD) method has been used for the numerical investigation of the designed structures. To verify the FDTD simulations, analytical formulas based on the transmission line method (TLM) have also been proposed for the initial structure.

The rest of this paper is organized as follows: The initial structure which is used to design the proposed biosensor structures is introduced in “Initial structure and its formulation”. Also, an analytical model is presented in this section to calculate the transmission spectrum of the initial structure. “Design of the proposed biosensor I” and “Design of the proposed biosensor II” introduce the first and second proposed biosensors. Biosensors application is explained in “Biosensors application”. The obtained results are summarized and compared with other works in “Discussions and comparisons”. Finally, the last section is devoted to conclusions.

## Initial structure and its formulation

**Figure 1:**
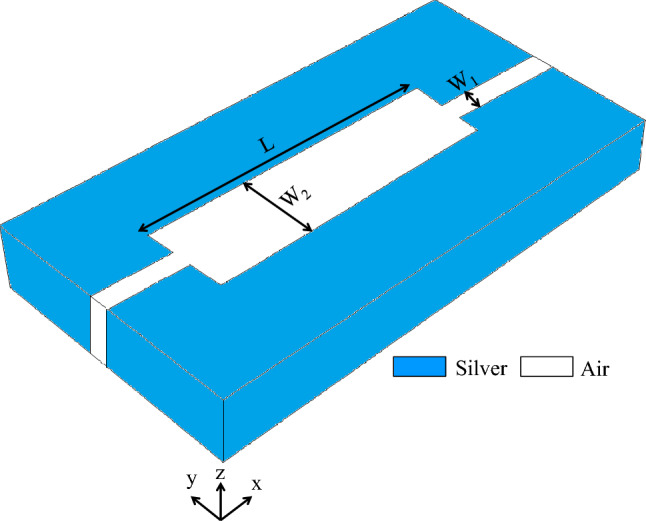
3D topology of the initial structure.

As shown in Fig. 1, a rectangular resonator connected to two MIM plasmonic waveguides is used to design the initial structure of the proposed sensors. The geometrical parameters of the initial structure include the length (L = 3980 nm) and width (W_2_ = 250 nm) of the rectangular resonator and the width of the MIM waveguide (W_1_ = 100 nm). The insulator layer is air with ${\varepsilon }_{d1}=1$, and the metal layers are silver. The complex relative permittivity of silver is characterized by the Drude model^59^:

1 $${\upvarepsilon }_{\mathrm{m}}\left(\upomega \right)= {\upvarepsilon }_{\infty }-\frac{{\upomega }_{\mathrm{p}}^{2}}{\upomega \left(\upomega +\mathrm{j \gamma }\right)},$$

where ε_∞_ = 3.7 is the medium dielectric constant for the infinite frequency, ω_p_ = 1.38 × 1016 Hz presents the bulk plasma frequency, ɤ = 2.73 × 1013 Hz denotes the electron collision frequency, and ω is the angular frequency of incident light.

The FDTD method is commonly used to model the MIM waveguide-based plasmonic structures^60,61^. Another method to model such structures is the TLM^62^. The second model, which is an analytical method, is usually used for the plasmonic structures with linear insulator materials. On the other hand, the FDTD method is more time-consuming than the TLM. Consequently, in addition to the FDTD method, the TLM has also been investigated for the initial structure.

**Figure 2:**
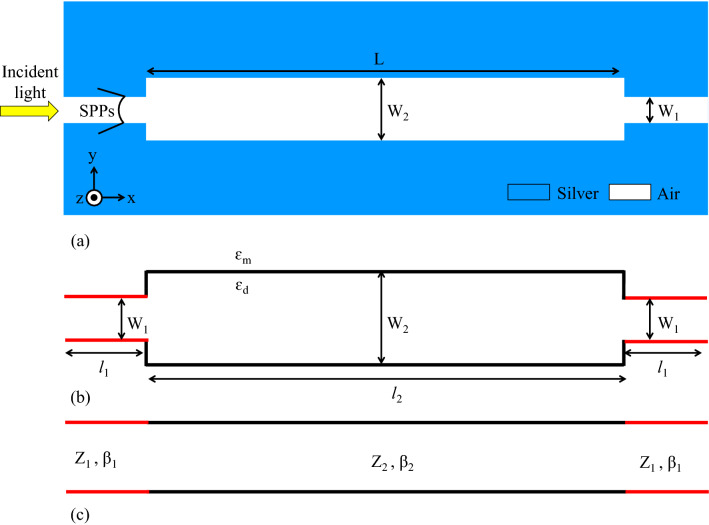
(**a**) 2D topology of the initial structure. (**b**) Schematic of MIM junctions between three waveguides, (**c**) Equivalent transmission line circuit of the initial structure.

After introducing the initial structure, the analytical model is presented to describe the behavior of this structure. Since the width of the rectangular resonator (W_2_) is close to the width of the MIM waveguides (W_1_) and $L\gg {W}_{2}$, the proposed initial structure can be considered as a combination of three cascaded waveguides with widths of W_1_, W_2_, and W_1_, respectively. Figure 2a shows the 2D topology of the initial structure. Also, the schematic of MIM junctions between three serial waveguides and the equivalent circuit of the initial structure are shown in Fig. 2b,c, respectively^9,30^. As seen in the equivalent transmission-line circuit (Fig. 2c), the MIM waveguides are modeled by semi-infinite transmission lines. In this model, the characteristic impedances of Z_1_ and Z_2_ are assigned to the waveguides with widths of W_1_ and W_2_, respectively. The microwave circuit theories can be used to obtain the values of characteristic impedances^63^. Accordingly, they are approximated by the ratio of the voltage to the current (Eq. 2):

2 $${Z}_{j}=\frac{{V}_{j}}{{I}_{j}}=\frac{\beta ({W}_{j}){W}_{j}}{\omega {\varepsilon }_{0}{\varepsilon }_{1}} j=1, 2,$$

where ${W}_{j}$ is the width of the waveguides, $\omega$ is the frequency of incident light, ${\varepsilon }_{0}$ is the linear dielectric constant, ${\varepsilon }_{1}$ the relative permittivity of the insulator, and $\beta$ is the propagation constant, calculated by Eq. (3)^64^:

3 $$\beta \left({W}_{j}\right)=k\bullet {n}_{eff}\left({W}_{j}\right) j=1, 2,$$

where $k$ can be given by Eq. (4)^64^:

4 $$k=\frac{2\pi }{\lambda }.$$

Here, the wavelength of λ is obtained by Eq. (5):

5 $$\lambda =\frac{2\pi c}{\omega },$$

where c is the light speed in vacuum. It is worth mentioning that ${n}_{eff}$ in Eq. (3) can be approximated by Eq. (6)^65^:

6 $${n}_{eff}=\sqrt{{\varepsilon }_{1}}{\left(1+\frac{\lambda }{\pi h\sqrt{{-\varepsilon }_{2}}}\sqrt{1+\frac{{\varepsilon }_{1}}{{-\varepsilon }_{2}}}\right)}^\frac{1}{2}.$$

**Figure 3:**
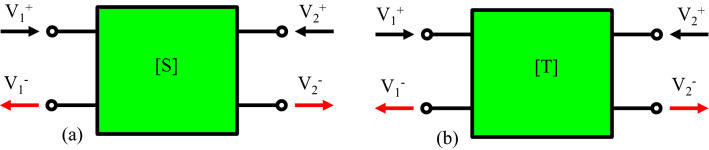
Schematic of relations between circuit input and outputs through (**a**) Scattering matrix. (**b**) Transfer matrix.

The transfer matrix method is used to calculate the transfer function of the transmission line circuit (Fig. 2c). Therefore, the scattering matrix of a plasmonic MIM junction is introduced (Fig. 3). The input and output voltages of Fig. 3 (${V}_{1}^{+}$, ${V}_{2}^{+}$, ${V}_{1}^{-}$, ${V}_{2}^{-}$) are given by Eqs. (7 and 8) ^66^:

7 $${V}_{1}\left(x\right)={V}_{1}^{+}{e}^{i\beta x}+{V}_{1}^{-}{e}^{-i\beta x},$$

8 $${V}_{2}\left(x\right)={V}_{2}^{+}{e}^{i\beta {x}^{^{\prime}}}+{V}_{2}^{-}{e}^{-i\beta {x}^{^{\prime}}},$$

where $x$ is the distance from the input port and ${x}^{^{\prime}}$ is the distance from the output port. Also, ${x+x}^{^{\prime}}$ presents the total distance from the input port to the output port. The voltages at the input and output ports of the line are related to each other by the scattering matrix of **S**^66^:

9 $$\left[\begin{array}{c}{\mathrm{V}}_{1}^{-}\\ {\mathrm{V}}_{2}^{-}\end{array}\right]=\mathbf{S}\left[\begin{array}{c}{\mathrm{V}}_{1}^{+}\\ {\mathrm{V}}_{2}^{+}\end{array}\right].$$

In this formula, the scattering matrix of a straight waveguide is given by^66^:

10 $${\mathbf{S}}_{MIM}=\left[\begin{array}{cc}0& {\mathrm{e}}^{-\mathrm{i\beta L}}\\ {\mathrm{e}}^{\mathrm{i\beta L}}& 0\end{array}\right].$$

Here, $L$ is the length of the straight waveguide. Furthermore, the scattering matrix of the direct junction of Fig. 2c is defined as^66^:

11 $${\mathbf{S}}_{jun}=\left[\begin{array}{cc}\Gamma & 1-\Gamma \\ 1+\Gamma & -\Gamma \end{array}\right].$$

In this formula, $\Gamma$ is calculated by:

12 $$\Gamma =\frac{{\mathrm{Z}}_{2}-{\mathrm{Z}}_{1}}{{\mathrm{Z}}_{2}+{\mathrm{Z}}_{1}}.$$

By introducing ${V}_{1}^{+,-}=\sqrt{{Z}_{1}}{ {\tilde{\text{V}}} }_{1}^{+,-}$ and ${V}_{2}^{+,-}=\sqrt{{Z}_{2}}{ {\tilde{\text{V}}} }_{2}^{+,-}$, the normalized scattering matrix can be given by:

13 $${\mathrm{S}}_{11}=-{\mathrm{S}}_{22}=\Gamma , {\mathrm{S}}_{12}={\mathrm{S}}_{21}=\frac{2\sqrt{{\mathrm{Z}}_{1}{\mathrm{Z}}_{2}}}{{\mathrm{Z}}_{1}+{\mathrm{Z}}_{2}}.$$

Here, ${{\tilde{\text{V}}}}_{1}^{+,-}$ and ${{\tilde{\text{V}}}}_{2}^{+,-}$ are the normalized input and output voltages, respectively. Thereafter, the transfer matrix of Fig. 3b can be calculated using the obtained scattering parameters^66^:

14 $$\left[\begin{array}{c}{\mathrm{V}}_{1}^{+}\\ {\mathrm{V}}_{1}^{-}\end{array}\right]=\mathbf{T}\left[\begin{array}{c}{\mathrm{V}}_{2}^{+}\\ {\mathrm{V}}_{2}^{-}\end{array}\right],$$

where $\mathbf{T}$ is given by:

15 $$\mathbf{T}=\frac{1}{{\mathrm{S}}_{21}}\left[\begin{array}{cc}1& -{\mathrm{S}}_{22}\\ {\mathrm{S}}_{11}& -\mathrm{Det}(\mathbf{S})\end{array}\right]=\left[\begin{array}{cc}{\mathrm{t}}_{11}& {\mathrm{t}}_{12}\\ {\mathrm{t}}_{21}& {\mathrm{t}}_{22}\end{array}\right].$$

In this formula, the matrix elements are defined as:

16 $${\mathrm{t}}_{11}=\frac{1}{2}\left(\sqrt{\frac{{Z}_{2}}{{Z}_{1}}}+\sqrt{\frac{{Z}_{1}}{{Z}_{2}}}\right) . {\mathrm{t}}_{12}=\frac{1}{2}\left(\sqrt{\frac{{Z}_{2}}{{Z}_{1}}}-\sqrt{\frac{{Z}_{1}}{{Z}_{2}}}\right).$$

Now, there are all the factors to calculate the total transfer matrix of the equivalent circuit of the initial structure. This transfer function can be given by:

17 $$\mathbf{T}={\mathbf{T}}_{1}\left({l}_{1}\right){\mathbf{T}}_{\mathrm{jun}\_1}{\mathbf{T}}_{2}\left({l}_{2}\right){\mathbf{T}}_{\mathrm{jun}\_2}{\mathbf{T}}_{1}\left({l}_{1}\right).$$

Here, the transfer matrixes of ${\mathbf{T}}_{j}\left({l}_{j}\right)$ and ${\mathbf{T}}_{jun\_j}$ are defined as:

18 $${\mathbf{T}}_{j}\left({l}_{j}\right)=\left[\begin{array}{cc}{\mathrm{e}}^{-i{\beta }_{j}{l}_{j}}& 0\\ 0& {\mathrm{e}}^{i{\beta }_{j}{l}_{j}}\end{array}\right]; j=1, 2,$$

19 $${\mathbf{T}}_{jun\_j}=\left[\begin{array}{cc}{\mathrm{t}}_{j}^{+}& {\mathrm{t}}_{j}^{-}\\ {\mathrm{t}}_{j}^{-}& {\mathrm{t}}_{j}^{+}\end{array}\right] , {\mathrm{t}}_{j}^{\pm }=\frac{\sqrt{\frac{{\mathrm{Z}}_{\mathrm{s}(\mathrm{i}+1)}}{{\mathrm{Z}}_{\mathrm{si}}}}\pm \sqrt{\frac{{\mathrm{Z}}_{\mathrm{si}}}{{\mathrm{Z}}_{\mathrm{s}(\mathrm{i}+1)}}}}{2}; j=1, 2.$$

Finally, the transfer function can be calculated by realizing the total transfer matrix (Eq. 20):

20 $$\mathrm{T}={\left|\frac{{\mathrm{V}}_{2}^{+}}{{\mathrm{V}}_{1}^{+}}\right|}^{2}.$$

**Figure 4:**
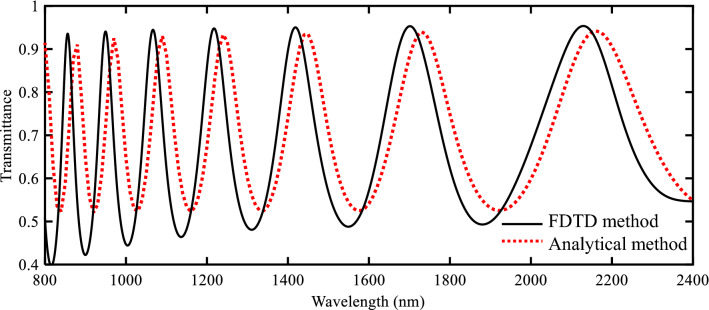
Transmission spectra of the initial structure using FDTD simulations and TLM.

After presenting the analytical model, the transmission spectrum of the initial structure is obtained using this method and compared with the FDTD method. Figure 4 shows these transmission spectra. As seen in this figure, good agreement can generally be shown between two methods. It is worth mentioning that there is a reason why these two curves do not match completely. This small amount of error stems from the fact that the approximated formula is used for the calculation of n_eff_ (Eq. 6).

## Design of the proposed biosensor I

**Figure 5:**
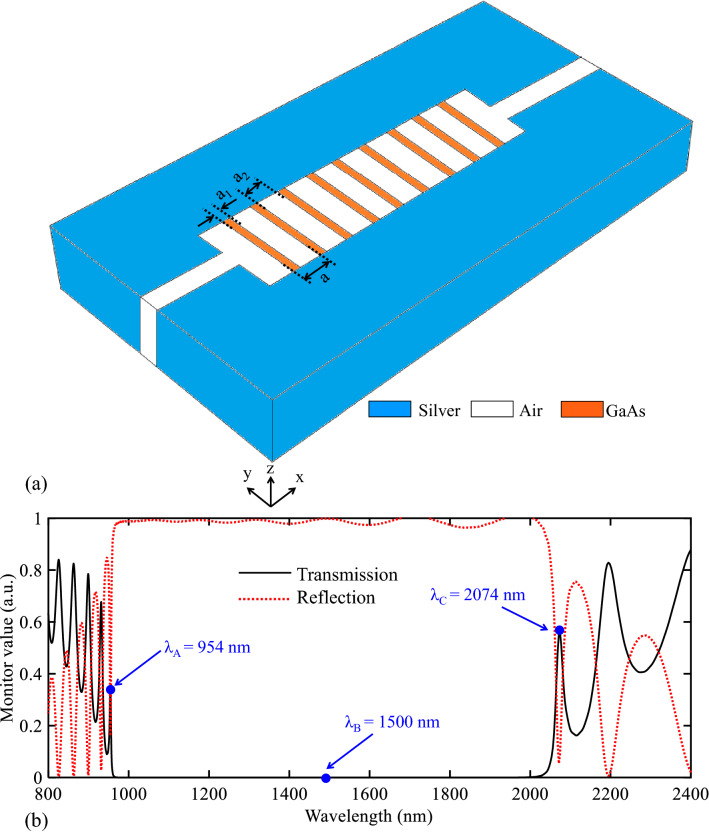
(**a**) 3D topology of the proposed biosensor I. (**b**) Its transmission and reflection spectra.

The initial structure of the proposed biosensors is presented in the previous section. The main problems of this structure are its multimode spectrum and low Q-factor modes. Accordingly, such a structure is not suitable for a sensor structure and it should be improved. In this section, the periodic GaAs (Palik model) insulator layers are inserted in the rectangular resonator to improve the initial structure. Figure 5a shows this structure. In this figure, the values of the geometrical parameters of “a_1_” and “a_2_” are equal to 80 and 330 nm. It is worth mentioning that the geometrical parameter of “a” is the lattice constant of the structure and it is equal to a = a_1_ + a_2_.

According to Fig. 5a, inserting the GaAs layers generates a 1D PC structure in the central part of the topology. Consequently, it can be expected that a PBG is generated in the transmission spectrum of this structure. Figure 5b shows the transmission and reflection spectra of the proposed biosensor I. As seen, this figure proves the claim of the existence of a PBG. The wavelength range of this PBG is extended from 954 to 2074 nm with sharp edges. The maximum transmission values of the PBG edges are equal to 33.8% and 56.1%, respectively. Therefore, the edges of this PBG can be used for sensing applications.

**Figure 6:**
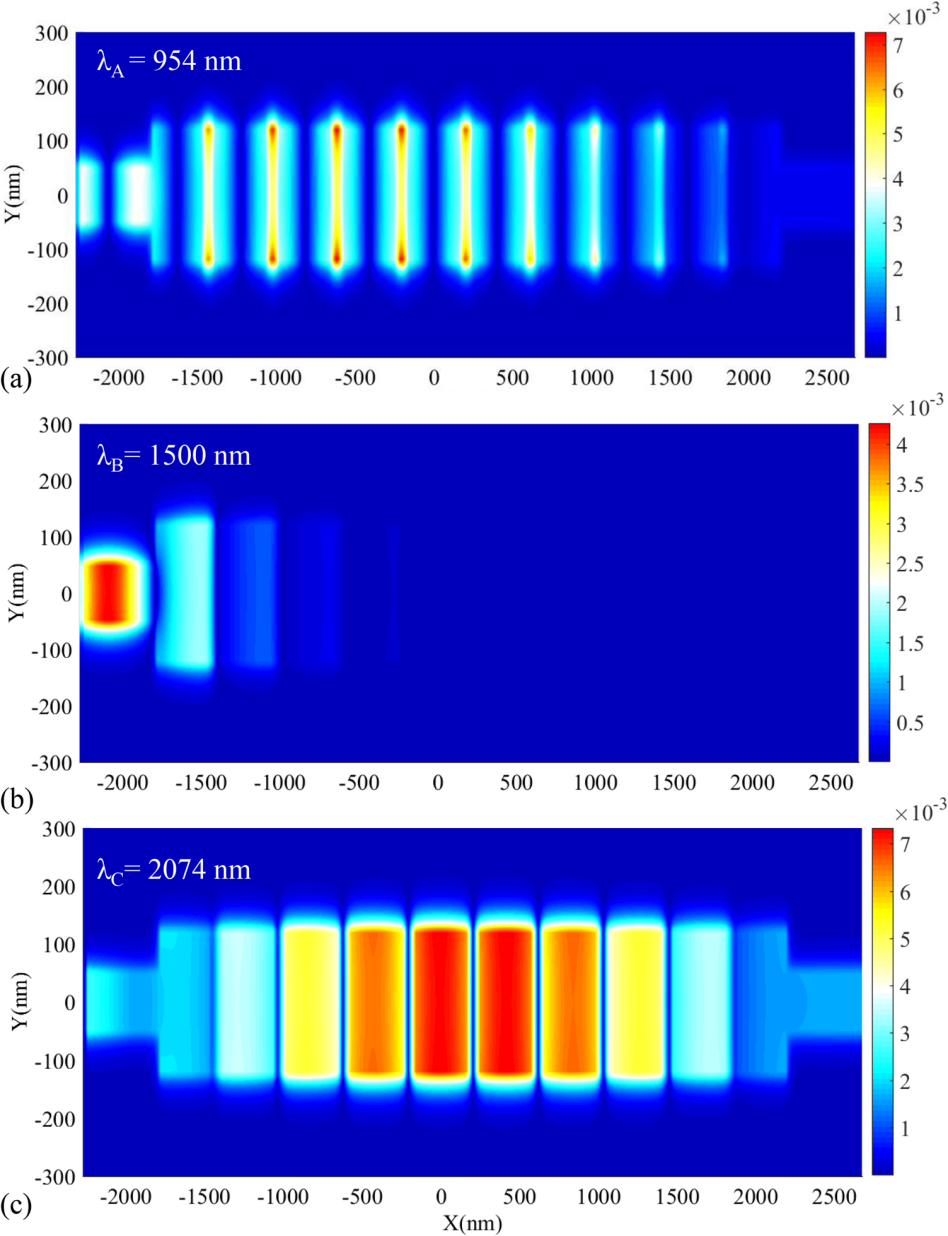
Field profile of $\left|{H}_{z}\right|$ for the proposed biosensor I at the wavelength of (**a**) λ_A_, (**b**) λ_B_, (**c**) λ_C_.

We also intend to provide a view of the operation mechanism of the proposed structure of biosensor I by its field profile. The field profile of $\left|{\mathrm{H}}_{\mathrm{z}}\right|$ for this structure is shown in Fig. 6. Figures 6a–c show the field profile of the proposed biosensor I at the wavelengths of λ_A_ = 954, λ_B_ = 1500, and λ_C_ = 2074 nm, respectively. As seen in Fig. 5a,c the wavelengths of λ_A_ and λ_C_ (wavelengths of the PBG’s edges) have appeared in the structure and are transmitted to the output port. Also, the proposed structure does not transmit the wavelength of λ_B_ which is located at the PBG region (Fig. 6b). It is because PCs can act as a perfect mirror and confine light in the PBG region.

**Figure 7:**
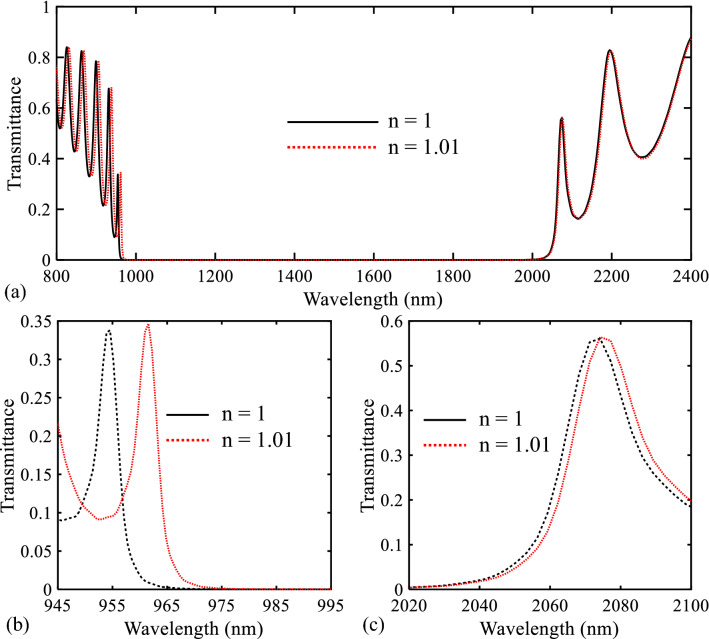
Transmission spectra of the proposed biosensor I for n = 1 and n = 1.01 in different wavelength ranges.

After designing the proposed biosensor I, the sensor operation has been investigated. The transmission spectrum variation of this structure for a 0.01 change in the refractive index of the analyte (air in this case) is shown in Fig. 7a. As seen, the transmission spectrum shifts to higher wavelengths by changing the refractive index of the analyte. Also, the zoomed views of the low and high PBG edges are shown in Fig. 7b,c, respectively. As seen in these figures, the wavelength shift of the low PBG is more than the high PBG. On the other hand, the sharpness of the low PBG edge is more the high PBG edge. It is also intended to design this structure for bio-optics applications whose wavelength is located at the NIR. As a result, the low PBG is more suitable for sensing applications.

**Figure 8:**
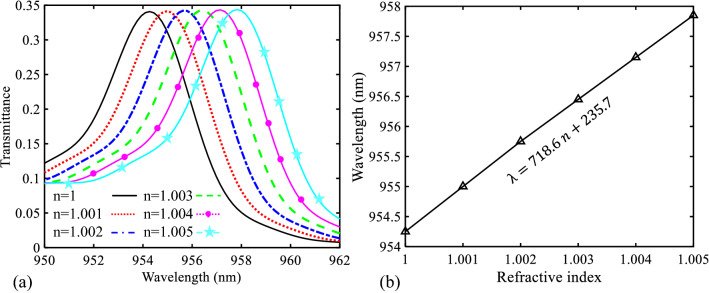
(**a**) Transmission spectra of the proposed biosensor I for refractive index changes from 1 to 1.005 in steps of 0.001. (**b**) Relationship between the wavelength of the PBG’s low edge and different values of the refractive index.

To provide a better view into the performance of the designed biosensor I, this structure is once again simulated for RI changes of 0.001. This case is shown in Fig. 8a. As seen, the transmittance curve shifts for the refractive index change step of 0.001 are quite clear so that this variation can be enough for sensing. Also, a linear function is fitted on the data points, to quantify the relationship between the refractive index increasing and the resonance wavelength shifting (Fig. 8b). As seen in this figure, the slope value of this curve is a large value (equal to 718.6 nm/RIU). Since this value shows the sensitivity of the proposed sensor I, the proposed structure I is a high sensitive sensor. It should be noted that the sensitivity value can be calculated by^67^:

21 $${S}_{\lambda }\left(\lambda \right)=\frac{\Delta \lambda }{\Delta n} \left(\frac{nm}{RIU}\right).$$

Based on the perturbation theory which is discussed in Refs.^22,42^, two parameters can increase the sensitivity of sensors. One of them is a high portion of the resonance mode energy (σ) and the other one is a high quality factor (Q-factor) value. The portion of the resonance mode energy for the proposed biosensor I has been studied in Fig. 6a by showing its field profile. As a result, it is desirable to calculate the Q-factor of the proposed structure. The Q-factor can be calculated by Eq. (22)^68^:

22 $$Q=\frac{{\lambda }_{res}}{\Gamma },$$

where ${\uplambda }_{\mathrm{res}}$ is the resonance wavelength and $\Gamma$ is the resonance bandwidth. For the resonance wavelength of the low PBG edge in the proposed biosensor I, the calculated Q-factor is equal to 207.4. The most comprehensive parameter that can be used for the comparison of the sensors’ operations is the figure of merit (FOM). It is because both factors of σ and Q-factor have been considered in this parameter. The FOM parameter can be calculated by Eq. (23)^42^:

23 $$\mathrm{FOM}=\frac{{\mathrm{S}}_{\uplambda }\left(\uplambda \right)}{\Gamma } \left({\mathrm{RIU}}^{-1}\right).$$

Base on Eq. (23), the calculated FOM value for the proposed biosensor I is 156.217 RIU^−1^.

**Figure 9:**
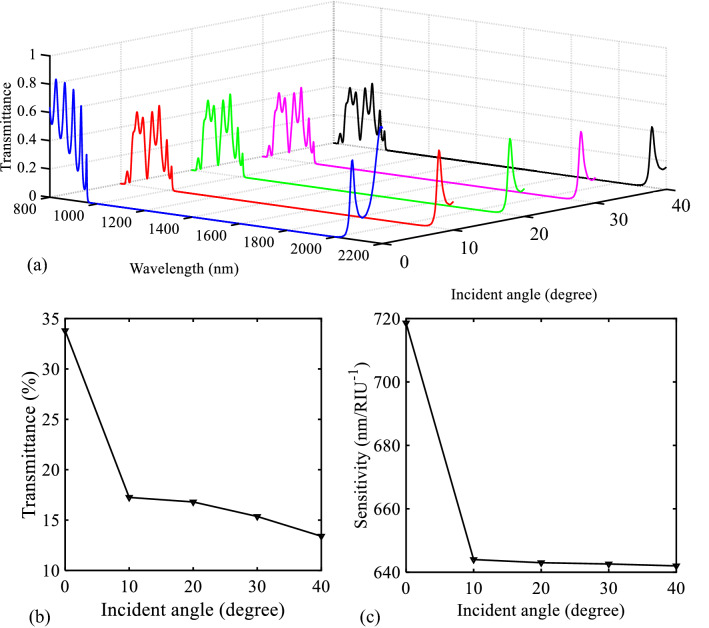
(**a**) Transmission spectra of the proposed biosensor I for different incident angles. (**b**) Relationship between the maximum transmission value of the low PBG edge and different values of the incident angle. (**c**) Relationship between the sensitivity value and different values of the incident angle.

After studying the sensing operation of biosensor I, some important issues such as the incident angle of the source light and the plasmonic effect on the sensor’s operation have been investigated. As shown in Fig. 2a (the initial structure), the incoming TM-polarized light irradiates one side of the sensor structure at an incidence angle θ = 0 degrees. In this part, we intend to investigate the effect of changing the incidence angle on the sensor’s performance. Figure 9a shows the transmission spectra of the proposed biosensor I for different values of incident angle of the light source. As seen in this figure, by deviating the incident angle from zero degrees, the PBG region shifts to higher wavelengths. Also, this change reduces the transmittance value of the low PBG edge and the sensitivity value of the proposed sensor structure. Figure 9b,c show these cases. Accordingly, the best choice for the incident angle is θ = 0 degrees.

**Figure 10:**
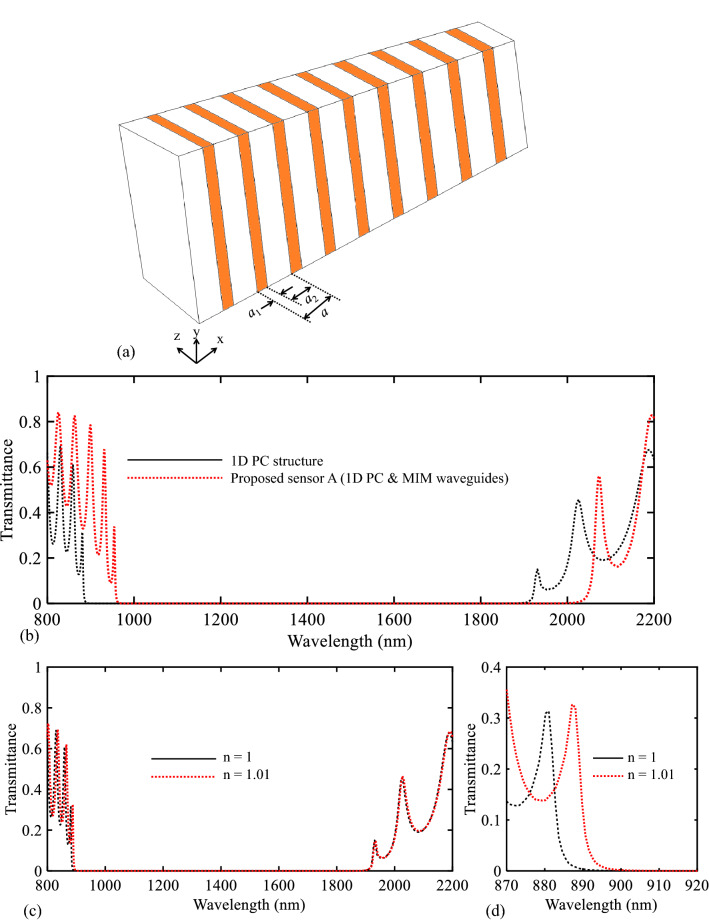
(**a**) 3D topology of the used PC structure in the proposed biosensor I. (**b**) Transmission spectra of the PC structure and biosensor I. (**c**,**d**) Transmission spectra of the proposed biosensor I for n = 1 and n = 1.01 in different wavelength ranges.

In order to investigate the plasmonic effect on the PBG and sensitivity of the proposed structure, the behavior of the PC structure without the plasmonic section has been investigated and compared to the proposed sensor structure. The 1D PC structure used in this paper is shown in Fig. 10a. The geometrical parameters of this topology have been already explained. In Fig. 10b, the transmission spectrum of the 1D PC structure obtained using the FDTD method has been compared to the transmission spectrum of the total proposed sensor topology. As seen in this figure, the plasmonic structure causes the PBG to shift to higher wavelengths with higher transmission values at its PBG’s edges which are more desirable.

By changing the refractive index of air layers from 1 to 1.01, the shift of its transmittance curve has also been investigated. Figure 10c,d show this case. As can be seen, the shift of the low PBG edge is equal to 6.1 nm. Based on the obtained results, the sensitivity value for the 1D PC is equal to 610 nm/RIU. Based on the obtained results, it can be concluded that the plasmonic structure also increases the sensitivity value of the proposed sensor. Accordingly, adding a plasmonic structure improves the sensing performance of the proposed structure.

## Design of the proposed biosensor II

**Figure 11:**
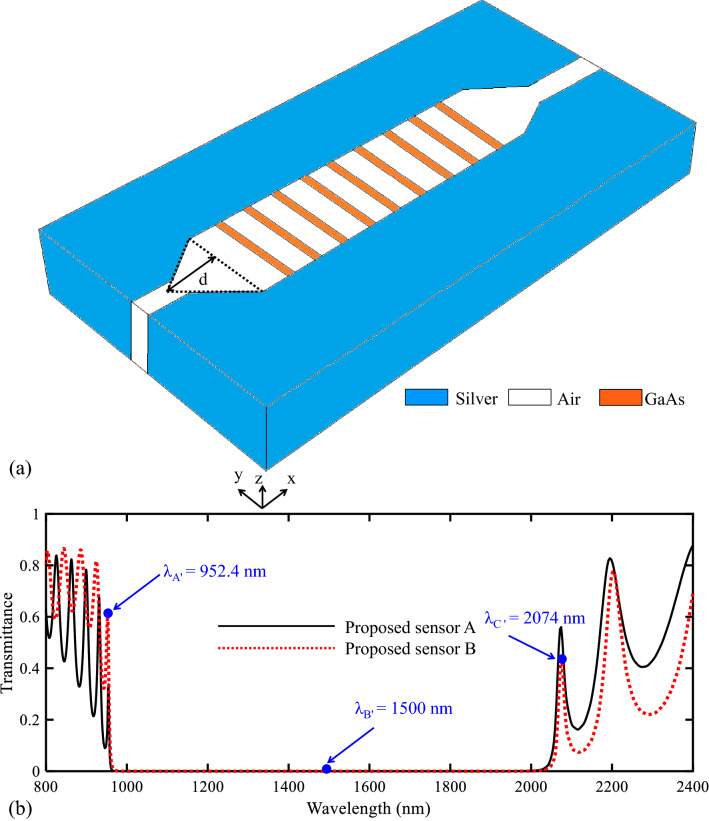
(**a**) 3D topology of the proposed biosensor II. (**b**) Transmission spectra of the proposed biosensor I and biosensor II.

To achieve higher transmittance values in the PBG’s edges, tapered resonators are added to the proposed biosensor I. The topology of the proposed biosensor II is shown in Fig. 11a. The value of the geometrical parameter of “d” is equal to 265 nm. Other parameters have been already explained. The transmission spectrum of the proposed biosensor II is shown in Fig. 11b and it is also compared with the transmission spectrum of the biosensor I. Figure 11b shows that the transmittance value in the low edge of the PBG (952.4 nm) increases (from 33.8 to 60.6%). Increasing the transmittance value in this PBG edge provides a biosensor with more transmittance value. It is because of the more coupling strength between MIM plasmonic waveguides and the central PC structure in this topology. As known, there is a trade-off between designing parameters of sensor structures. Increasing the coupling effect between MIM plasmonic waveguides and PC leads to slower sharpness in transition from the maximum transmittance to the minimum transmittance. Accordingly, it can be caused that the sensitivity of biosensor II is slightly lower than the sensitivity of biosensor I.

**Figure 12:**
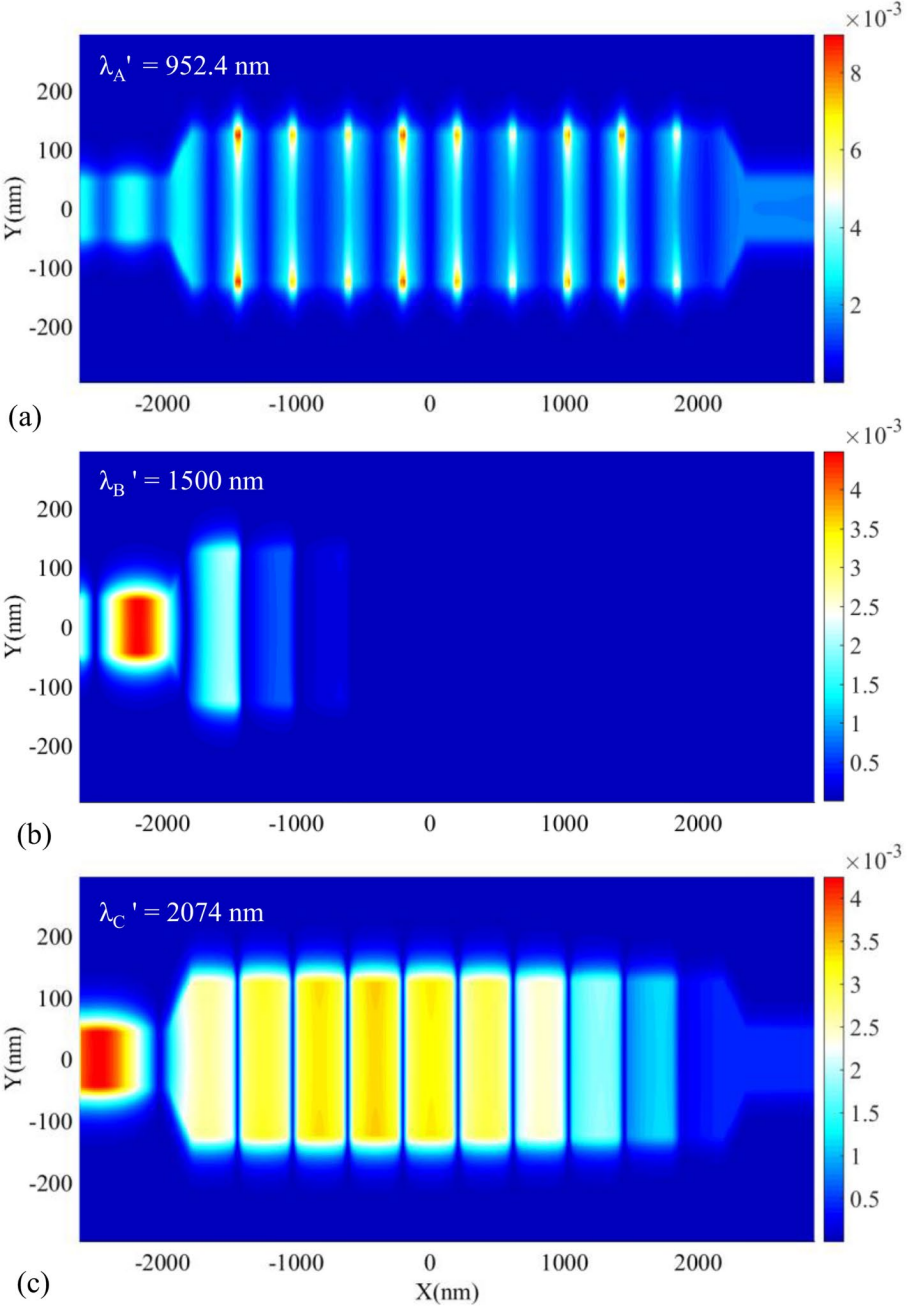
Field profile of $\left|{H}_{z}\right|$ for the proposed biosensor II at the wavelength of (**a**) λ_A'_, (**b**) λ_B'_, (**c**) λ_C'_.

Due to the appropriate view of the magnitude of H_z_, the field profile of $\left|{\mathrm{H}}_{\mathrm{z}}\right|$ for the proposed biosensor II has also been investigated. Figure 12 shows this case. As seen in Fig. 12a,c, the incident light at the wavelengths of the PBG’s edges (λ_A'_ = 952.4 and λ_C'_ = 2074 nm) can pass through the structure. Also, Fig. 12b shows the field profile of $\left|{\mathrm{H}}_{\mathrm{z}}\right|$ for λ_B'_ = 1500 nm, which cannot be transmitted to the output port. It is because this wavelength is located at the PBG region.

**Figure 13:**
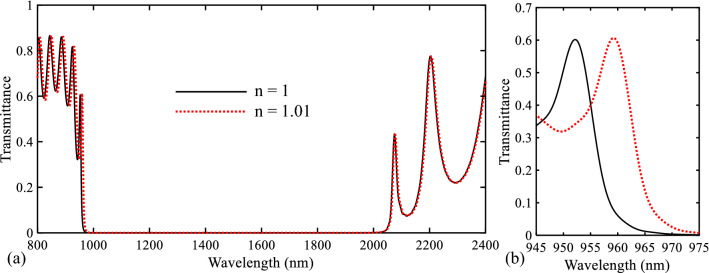
Transmission spectra of the proposed biosensor II for n = 1 and n = 1.01 in different wavelength ranges.

To verify the operation mechanism of the proposed biosensor II, the shift of its transmittance curve has also been investigated by changing the refractive index of the analyte from 1 to 1.01. Figure 13a shows this change in the transmittance curve of biosensor II. As expected, a high-frequency shift occurs in the low PBG’s edge, while the high PBG’s edge experiences a little frequency shift. The zoomed view of the low PBG's edge is shown in Fig. 13b. As seen in this figure, the shift of the low PBG’s edge is equal to 7.143 nm. It is worth mentioning that the obtained sensitivity, Q-factor, and FOM values for the proposed biosensor II are 714.3 nm/RIU^−1^, 80.16, and 60.1 RIU^−1^, respectively.

**Figure 14:**
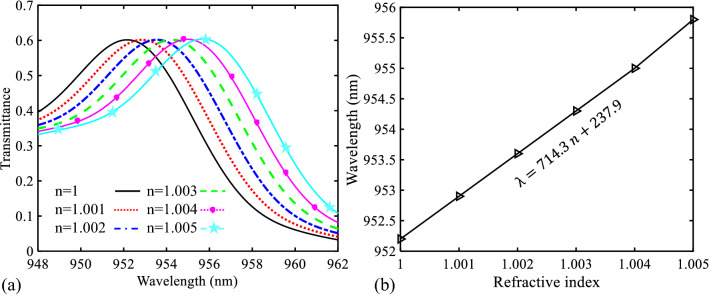
(**a**) Transmission spectra of the proposed biosensor II for refractive index changes from 1 to 1.005 in steps of 0.001. (**b**) Relationship between the wavelength of the PBG’s low edge and different values of the refractive index.

Similar to the previous sensor structure (biosensor I), the shift of the transmittance curve for biosensor II is considered by changing the refractive index of its analyte from 1 to 1.01 in steps of 0.001. Figure 14a shows these changes. The relationship between the refractive index increasing and the resonance wavelength shifting is also shown in Fig. 14b. The fitted linear function on the data points of this figure shows that the slope of this curve is lower than the previous case (Fig. 8b). This difference is not too much.

**Figure 15:**
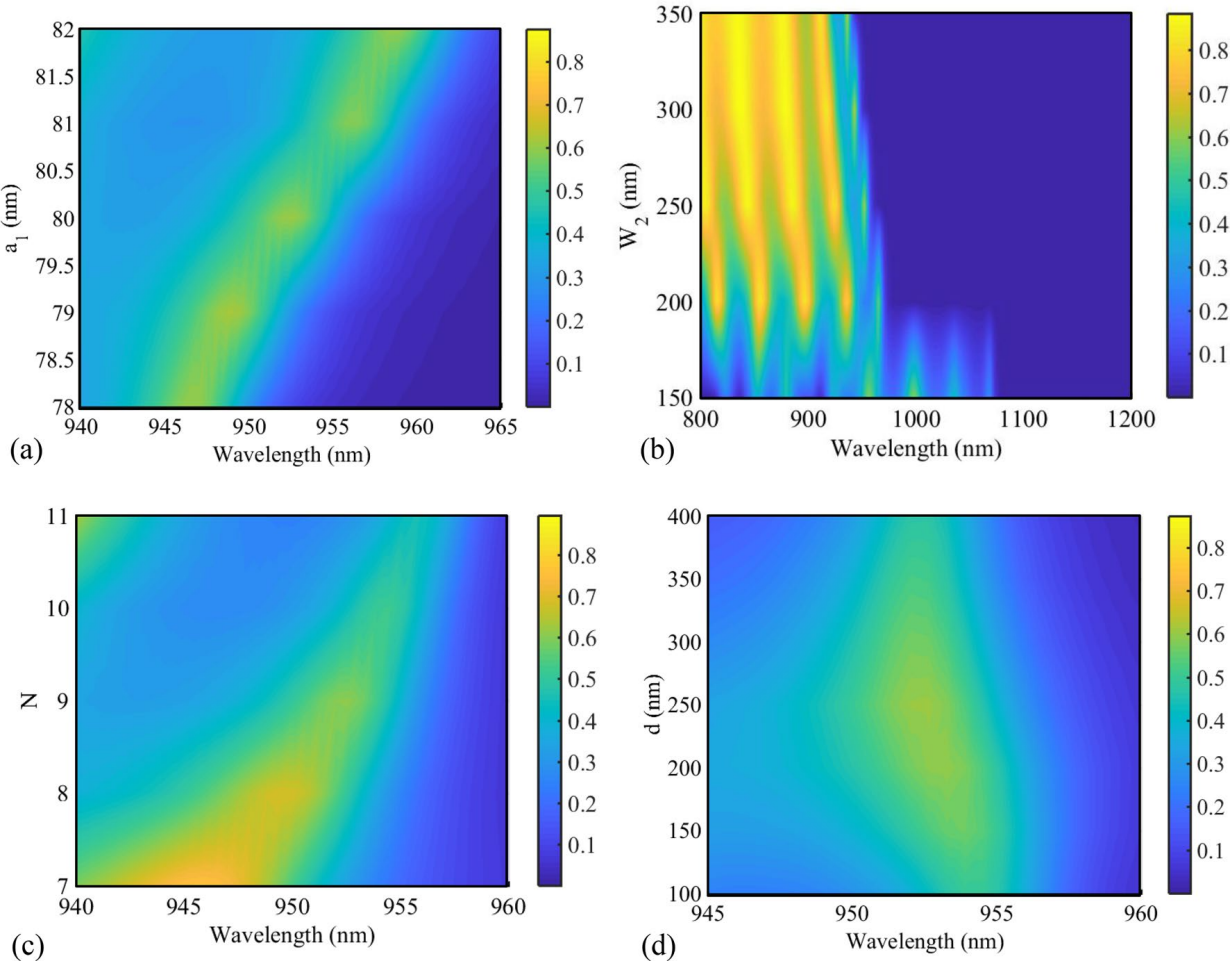
Transmission spectra of the proposed biosensor II for different values of (**a**) a_1_, (**b**) W_2_, (**c**) N, and (**d**) d.

To investigate the effect of some geometrical parameters of the proposed biosensor II on its transmission spectrum, the “a_1_”, “W_2_”, “number of the GaAs layers (N)”, and “d” parameters have been swept. The first three parameters are related to the 1D PC structure, and the last parameter is related to tapered resonators. As seen in Fig. 15a, by increasing the “a_1_” value from 78 to 82 nm, the low PBG edge shifts to higher wavelengths. Consequently, by changing the geometrical parameter of “a_1_”, the wavelength of the low PBG edge can be tuned. The transmission spectra of the proposed biosensor II as a function of W_2_ are also shown in Fig. 15b. As seen in this figure, increasing W_2_ corresponds to a lower sensing wavelength (the wavelength of the low PBG edge). Thereafter, the number of the GaAs layers is changed from 7 to 11 layers (Fig. 15c). It can be seen that increasing “N” shifts the low PBG edge to higher wavelengths. Also, Fig. 15d shows the transmission spectra of biosensor II for different values of “d”. As seen in this figure, when the value of “d” is increased, the location of the low PBG edge is almost constant.

**Figure 16:**
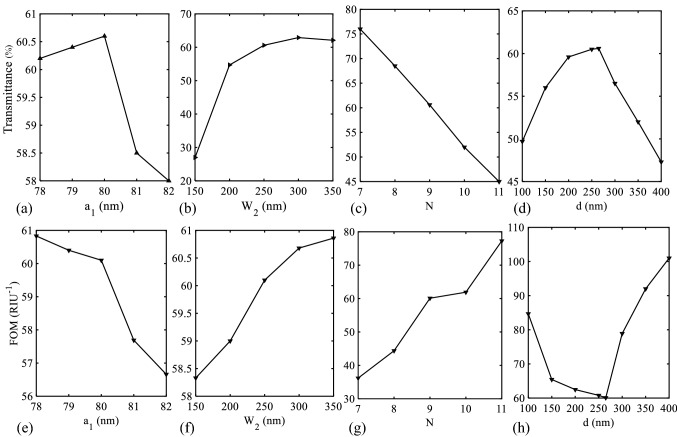
Relationship between the maximum transmission value of the low PBG edge and different values of (**a**) a_1_, (**b**) W_2_, (**c**) N, and (**d**) d. Relationship between the FOM value of the proposed biosensor II and different values of (**e**) a_1_, (**f**) W_2_, (**g**) N, and (**h**) d.

At the next step, the important characteristic values of the biosensor II (transmittance value of the low PBG edge and FOM) for different values of “a_1_”, “W_2_”, “N”, and “d” have also been investigated to provide a better view of how such geometrical parameters changes affect the operation of the biosensor II. Figure 16 shows these changes. Figure 16a,e show that by increasing the “a_1_” value, the transmittance value increases initially and then decreases, and the FOM value is almost constant from a_1_ = 78 to 80 nm and then decreases. Accordingly, the value of 80 nm (with the highest transmittance value and a relatively high FOM value) is the best choice. Another parameter whose variation has been investigated is the “W_2_” parameter (Fig. 16b,f). As seen, increasing the “W_2_” value corresponds to higher transmittance and FOM values. On the other hand, the total size of the biosensor II increases by increasing the “W_2_” value. It should be that there is a trade-off between designing parameters of a sensor structure. Consequently, the medium value of 250 nm has been selected for the “W_2_” parameter.

The next parameter is the number of GaAs layers (Fig. 16c,g). As seen, when the “N” parameter is increased, the transmittance and FOM values decrease and increase, respectively. Similar to the previous parameter (“W_2_” parameter), increasing “N” causes the total size of the biosensor II to increase. Accordingly, to create a trade-off between different design parameters, the value of N = 9 has been selected. Finally, the last parameter is “d” (Fig. 16d,h). As seen in Fig. 16d, by increasing “d”, the transmittance value increases initially and then decreases. The highest transmittance value occurs at d = 265 nm. On the other hand, such variations of the “d” parameter cause the FOM value to decrease initially and then increase. As discussed before, the purpose of adding tapered resonators is to increase the transmittance value. Because the proposed biosensor I has a high FOM value. Therefore, the value of 265 nm, which corresponds to the highest transmittance value, has been chosen to design the proposed biosensor II.

## Biosensors application

**Figure 17:**
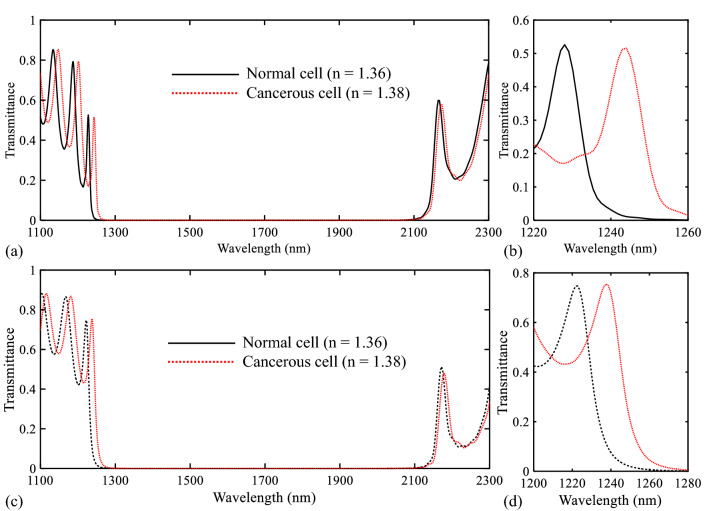
(**a**,**b**) Transmission spectra of the proposed biosensor I for normal (n = 1.36) and cancerous (n = 1.38) cells in different wavelength ranges. (**c**,**d**) Transmission spectra of the proposed biosensor II for normal (n = 1.36) and cancerous (n = 1.38) cells in different wavelength ranges.

After reviewing the performance of the designed sensor structures using air as the insulator material of the analyte, their behavior is also investigated for a special application. One of the attractive subjects in bio-optics is measuring the tissues’ refractive index. In this section, it is shown that the proposed structures can be used for the detection of basal cell cancer. It is because the used wavelengths of the proposed sensors (the PBGs’ low edges) are located at the NIR. On the other hand, in Ref.^69^, the refractive index of human cells has been comprehensively measured and reported at this frequency range. The human skin tissue can be modeled using a mixture of water and organic compounds. Because this tissue is composed of approximately 70% water and 30% protein^69^. The refractive indices of different sections of a cell for NIR are estimated as follows: cytoplasm: 1.36–1.375, extracellular fluid: 1.35–1.36, nucleus: 1.38–1.41, and melanin: 1.6–1.7. Since cancerous cells have more protein in their cytoplasm, they have a higher refractive index value^70^. The refractive indices of the cytoplasm for normal and cancerous basal cells are equal to 1.36 and 1.38, respectively^70^. Figure 17 shows the transmission spectra of the proposed biosensors for these refractive indices (refractive indices of normal and cancerous cells). As seen in this figure, the wavelengths of low PBGs’ edges are steel located at the NIR for the refractive indices of normal and cancerous cells. Consequently, the proposed structures can be easily used for the detection of basal cell cancer. Also, enough contrast between normal and cancerous cells in both cases causes the proposed sensors can be good candidates for this application.

## Discussions and comparisons

As mentioned before, the FDTD method has been used to design the proposed structures. Also, the fabrication procedure of the proposed structures is similar to what is discussed in Ref.^71^. It is worth mentioning that there are some potential challenges in their fabrication process. The first one is the efficient coupling of light to the plasmonic waveguides. Using gratings or the Kretschmann prism configurations is usually suggested for this challenge. The tarnishing of silver which changes its refractive index is the second challenge. Accordingly, the fabrication procedure should be performed in a chamber emptied from air.

To provide a better view of the obtained results, the proposed biosensors have been compared with other reported works in recent years. Table 1 shows some main characteristics of sensors for comparison. In this table, the year of the papers’ publication, the topology type of the sensors, the spectrum type of their transmission spectra, sensing wavelengths (${\lambda }_{r}$), the Q-factor, sensitivity, and FOM have been compared.

**Table 1:** Performance comparisons between the proposed sensors and other works.

References	Year	Topology	Spectrum	λ_r_ (nm)	Q-factor	Sensitivity (nm/RIU)	FOM (RIU^−1^)
Ref.^22^	2021	Plasmonic	Lorentzian	592	304.06	550	282.5
Ref.^23^	2021	Plasmonic	Lorentzian	988.8	132.8	1000	133
Ref.^42^	2021	Plasmonic	Lorentzian	1056.4	69.91	1050	69.5
Ref.^44^	2021	Plasmonic	Lorentzian	794	–	400	–
Ref.^72^	2021	Plasmonic	Fano resonance	839	–	795	–
Ref.^73^	2019	Plasmonic	Lorentzian	808	269.3	636	211.3
Ref.^74^	2017	Plasmonic	EIT-like	562.7	–	520	178
Ref.^75^	2018	PC	Lorentzian	1309	–	720	–
Ref.^75^	2018	PC	Lorentzian	1309	–	638	–
Biosensor I	–	PC and plasmonic	PBG	954	207.4	718.6	156.217
Biosensor II	–	PC and plasmonic	PBG	952.4	80.16	714.3	60.1

As seen in this table, the topology type of some published works is plasmonic structure^22,23,42,44,72–74^ and the topology type of some others is PC^75^. As discussed before, each of these topologies has some advantages and some disadvantages. In this paper, in order to benefit from the advantages of both topologies, the combination of them has been used. Another comparison parameter is the spectrum type of the transmission spectra. As seen, most of them have a Lorentzian spectrum^22,23,42,44,73,75^. Although such a spectrum has some advantages such as symmetrical shape, it has some drawbacks for sensing applications. The first one is that their edges cannot be very sharp. It is because this issue may lead to fabrication errors in real experimental situations. On the other hand, most of these spectra are multi-mode, while using multi-mode spectra for designing sensors is not desirable. It is because these modes may interfere with each other by changing the refractive index of their analytes. Other types of spectra that have been used for sensing applications are Fano-resonance^72^ and EIT-like resonance^74^. In this paper, the PBG transmission spectrum, which is less common in other published works, is used for sensing applications. This type of spectrum has different advantages. First, the sharp edges of PBGs are good candidates for sensing. The second one is that there is a long distance between two PBG edges. This causes that when the low PBG is used for sensing, it does not have an overlapping with the high PBG edge by changing the refractive index of the analyte. For this reason, the proposed structure can be used for a wide wavelength range. The other parameter is the sensing wavelength. As seen, the sensing wavelengths of the proposed sensors are located in the NIR which are suitable to use for biosensing applications. The other parameter is Q-factor. As seen, the Q-factors of the proposed structures are neither very low nor very high. It is because the low Q-factor decreases the FOM and the high Q-factor increases the effect of lithography error in the fabrication process. The last parameters are sensitivity and FOM. As discussed before, the most comprehensive parameter for the comparison of sensors’ operation is the FOM parameter. For example, the sensor designed in Ref.^42^ has the most sensitivity in the comparison table, while its FOM value is low. As seen, the proposed biosensor I is among the highest FOM sensors.

## Conclusion

In this paper, two novel biosensors based on a 1D PC and plasmonic structures were proposed. The combination of PC and plasmonic structures causes a suitable balance between different designing parameters can be obtained. The FDTD simulation has been used for numerical investigation of the designed structures. The obtained results show that the sensitivity values of 718.6 and 714.3 nm/RIU have been achieved for the designed biosensors I and II, respectively. The sharp PBG edges of the biosensors’ transmission spectra result in high sensitivity detections. The proposed structures could find potential for bio-optical sensing applications such as cancer cell detection.

## Data Availability

The calculated results during the current study are available from the corresponding author on reasonable request.
